# Upright BPPV Protocol: Feasibility of a New Diagnostic Paradigm for Lateral Semicircular Canal Benign Paroxysmal Positional Vertigo Compared to Standard Diagnostic Maneuvers

**DOI:** 10.3389/fneur.2020.578305

**Published:** 2020-11-19

**Authors:** Salvatore Martellucci, Pasquale Malara, Andrea Castellucci, Rudi Pecci, Beatrice Giannoni, Vincenzo Marcelli, Alfonso Scarpa, Ettore Cassandro, Silvia Quaglieri, Marco Lucio Manfrin, Elisabetta Rebecchi, Enrico Armato, Francesco Comacchio, Marta Mion, Giuseppe Attanasio, Massimo Ralli, Antonio Greco, Marco de Vincentiis, Cecilia Botti, Luisa Savoldi, Luigi Califano, Angelo Ghidini, Giulio Pagliuca, Veronica Clemenzi, Andrea Stolfa, Andrea Gallo, Giacinto Asprella Libonati

**Affiliations:** ^1^ENT Unit, Santa Maria Goretti Hospital, AUSL Latina, Latina, Italy; ^2^Audiology & Vestibology Service, Centromedico Bellinzona, Bellinzona, Switzerland; ^3^ENT Unit, Department of Surgery, Azienda USL-IRCCS di Reggio Emilia, Reggio Emilia, Italy; ^4^Audiology Unit, AOU Careggi, Department of Surgery and Translational Medicine, University of Florence, Florence, Italy; ^5^A.S.L. Napoli 1 Centro, Ospedale del Mare, Naples, Italy; ^6^Department of Medicine and Surgery, University of Salerno, Salerno, Italy; ^7^ENT Unit, Policlinico San Matteo Fondazione (IRCCS), Pavia, Italy; ^8^ENT Unit, Guglielmo da Saliceto Hospital, Piacenza, Italy; ^9^ENT Unit, SS Giovanni e Paolo Hospital, Venice, Italy; ^10^Department of Neurosciences, Regional Specialized Vertigo Center, Institute of Otolaryngology, University of Padua, Padua, Italy; ^11^ENT Unit, Department of Surgery, Santa Maria Della Misericordia Hospital, Rovigo, Italy; ^12^Head and Neck Department, ENT Clinic, Policlinico Umberto I, Rome, Italy; ^13^Department of Sense Organs, Sapienza University of Rome, Rome, Italy; ^14^Department of Oral and Maxillofacial Sciences, Sapienza University of Rome, Rome, Italy; ^15^PhD Program in Clinical and Experimental Medicine, University of Modena and Reggio Emilia, Modena, Italy; ^16^Department Infrastructure Research and Statistics, Azienda USL-IRCCS di Reggio Emilia, Reggio Emilia, Italy; ^17^Departmental Unit of Audiology and Phoniatrics, G. Rummo Hospital Group, Benevento, Italy; ^18^Vestibology and ENT Unit, Giovanni Paolo II Hospital, Matera, Italy

**Keywords:** BPPV, horizontal semicircular canal BPPV, upright head roll test, lateral semicircular canal BPPV, head pitch test, upright BPPV protocol

## Abstract

**Background:** The diagnosis of benign paroxysmal positional vertigo (BPPV) involving the lateral semicircular canal (LSC) is traditionally entrusted to the supine head roll test, also known as supine head yaw test (SHYT), which usually allows identification of the pathologic side and BPPV form (geotropic vs. apogeotropic). Nevertheless, SHYT may not always allow easy detection of the affected canal, resulting in similar responses on both sides and intense autonomic symptoms in patients with recent onset of vertigo. The newly introduced upright head roll test (UHRT) represents a diagnostic maneuver for LSC-BPPV, supplementing the already-known head pitch test (HPT) in the sitting position. The combination of these two tests should enable clinicians to determine the precise location of debris within LSC, avoiding disturbing symptoms related to supine positionings. Therefore, we proposed the upright BPPV protocol (UBP), a test battery exclusively performed in the upright position, including the evaluation of pseudo-spontaneous nystagmus (PSN), HPT and UHRT. The purpose of this multicenter study is to determine the feasibility of UBP in the diagnosis of LSC-BPPV.

**Methods:** We retrospectively reviewed the clinical data of 134 consecutive patients diagnosed with LSC-BPPV. All of them received both UBP and the complete diagnostic protocol (CDP), including the evaluation of PSN and data resulting from HPT, UHRT, seated-supine positioning test (SSPT), and SHYT.

**Results:** A correct diagnosis for LSC-BPPV was achieved in 95.5% of cases using exclusively the UBP, with a highly significant concordance with the CDP (*p* < 0.000, Cohen's kappa = 0.94), regardless of the time elapsed from symptom onset to diagnosis. The concordance between UBP and CDP was not impaired even when cases in which HPT and/or UHRT provided incomplete results were included (*p* < 0.000). Correct diagnosis using the supine diagnostic protocol (SDP, including SSPT + SHYT) or the sole SHYT was achieved in 85.1% of cases, with similar statistical concordance (*p* < 0.000) and weaker strength of relationship (Cohen's kappa = 0.80).

**Conclusion:** UBP allows correct diagnosis in LSC-BPPV from the sitting position in most cases, sparing the patient supine positionings and related symptoms. UBP could also allow clinicians to proceed directly with repositioning maneuvers from the upright position.

## Introduction

Benign paroxysmal positional vertigo (BPPV) involving the lateral semicircular canal (LSC) is the second most common subtype of BPPV, accounting for <15% of all BPPV (1–3). It accounts for vertigo spells provoked by head position changes in the sitting and supine positions, and it is accompanied by positional and direction-changing horizontal nystagmus elicited by turning the head to either side (4, 5).

Canalolithiasis and cupulolithiasis are the most commonly accepted pathomechanisms underlying LSC-BPPV (4, 5). In canalolithiasis, free-floating otoliths within the canal can modify cupula sensitivity to accelerations, whereas in cupulolithiasis, debris are attached to the cupula overloading the cupula itself. In both cases, the cupula becomes sensitive to linear accelerations such as gravity and linear vectorial components induced by brisk head movements aligning with the plane of the involved canal (4–10).

The most widely used diagnostic test for LSC-BPPV is the supine head roll test, also named the “McClure–Pagnini maneuver” (1, 2, 11, 12), consisting of turning the patient's head 180° to either side while supine. Since it is performed along the yaw plane, it should be most properly called the “supine head yaw test” (SHYT) (13).

Depending on the direction of nystagmus evoked by SHYT, two variants of LSC-BPPV can be distinguished. In geotropic form, the paroxysmal nystagmus beats horizontally toward the undermost ear in both sides, since free-moving debris gravitate along the posterior arm of LSC toward the ampulla, thus exciting the ampullary receptor (1–13). Conversely, in an apogeotropic variant, particles can settle in the ampullary arm of the canal or adhere to the cupula, resulting in either paroxysmal or persistent nystagmus, respectively, beating toward the uppermost ear, as resulting endolymphatic displacement is ampullofugal, thus inhibiting the afferent resting firing rate (1–13).

Detection of the affected ear and involved arm is pivotal for successful repositioning. The first clinical sign used for the diagnosis of the affected side was nystagmus amplitude evoked by SHYT. In accordance with Ewald's second law, postulating that excitatory responses prevail over inhibitory outputs, the nystagmus elicited by SHYT beats with greater intensity toward the impaired ear compared to the contralateral side. Therefore, the affected side is where the nystagmus is more intense in geotropic variants, whereas the involved ear is the side where the nystagmus is weaker in apogeotropic forms (1, 2, 4–8).

Nevertheless, the diagnosis of the affected ear using the sole SHYT could be challenging, as differences in nystagmus amplitude and intensity could sometimes be hardly detected, despite the use of a video-oculography system possibly helping in this task (14). Additionally, keeping repeating SHYT could further reduce its sensitivity as nystagmus intensity may be altered by fatigability, and patients with recent onset of BPPV may experience significant discomfort and intense autonomic symptoms, potentially impeding diagnosis and treatment.

Some additional signs of laterality, listed as “secondary signs of lateralization” (14), could be sought to determine the precise location of debris in LSC-BPPV (14–28). These signs were firstly systematized into a diagnostic algorithm known as “minimum stimulus strategy” (MSS), with the aim to analyze changes in direction and/or intensity of the nystagmus as a function of head positions in space (13, 15, 29). MSS represents a three-step algorithm performed with the aid of video Frenzel goggles to monitor the plane and direction of the nystagmus (nystagmus-guided approach), aiming to result in the lowest discomfort possible.

It includes, as the first step, the evaluation of pseudo-spontaneous nystagmus (PSN) and nystagmus behavior during the head pitch test (HPT) performed in the sitting position. PSN is purely horizontal and differs from the direction-fixed nystagmus as its direction changes according to the head-bending angle (13, 15–18). HPT (or bow-and-lean test) consists of changing the angle between LSC and the horizontal plane by moving the patient's head along the pitch plane. During neck flexion, geotropic forms result in ampullopetal endolymphatic flows, evoking nystagmus beating toward the affected side, whereas resulting ampullofugal endolymphatic flows generate nystagmus toward the healthy ear in apogeotropic variants. Conversely, during neck extension, HPT determines reversed endolymphatic flows resulting in nystagmus beating opposite to previously reported movements (13–15, 19–22, 29).

**Figure 1:**
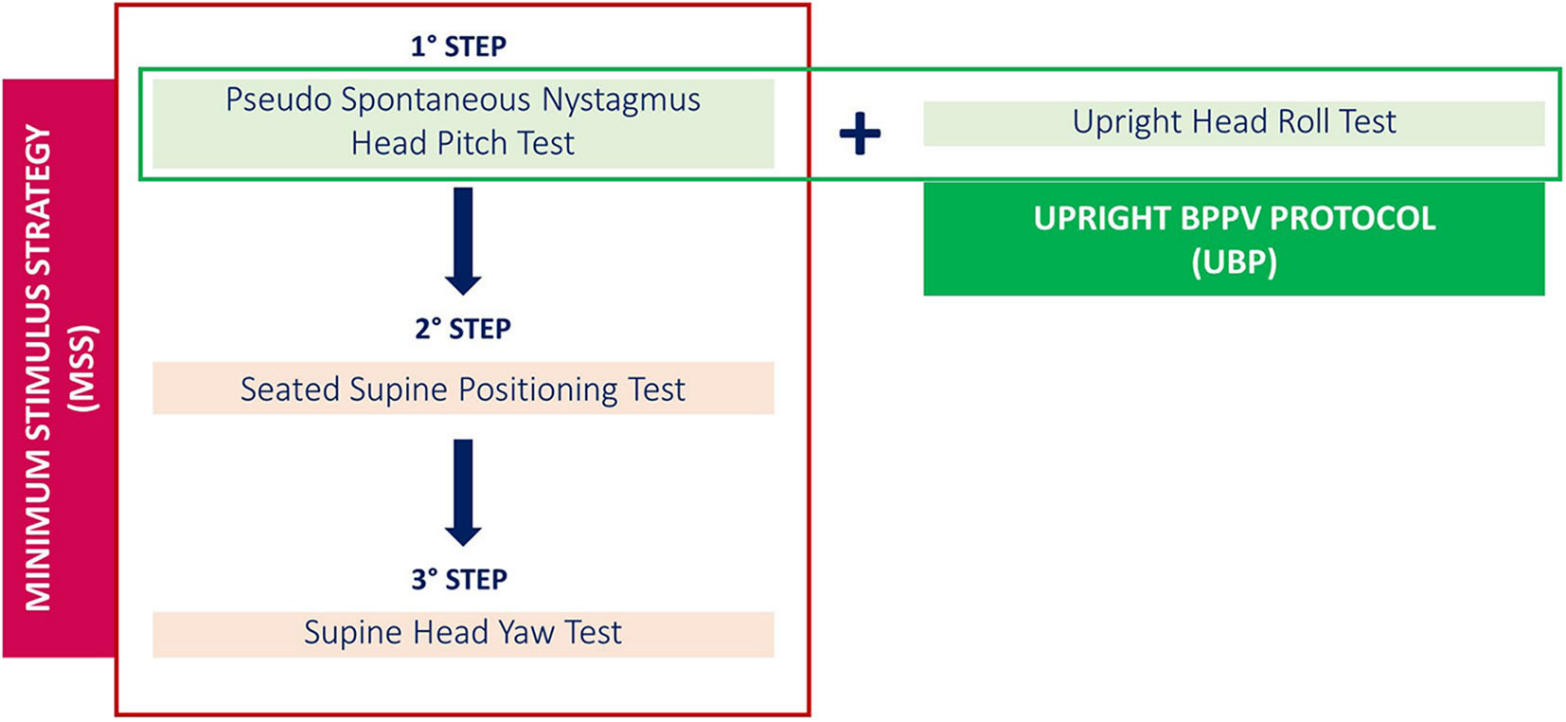
Diagnostic algorithm for LSC-BPPV: minimum stimulus strategy (MSS) and upright BPPV protocol (UBP).

The second step of MSS is the seated-supine positioning test (SSPT or lying-down test), consisting of bringing the patient down from the sitting to the supine positions. In LSC-BPPV, SSPT should evoke nystagmus beating toward the unaffected ear in geotropic forms and toward the opposite side in apogeotropic forms (8, 13, 15, 23–27, 29). Finally, the third and last step of MSS is SHYT. MSS is described in Figure 1.

The upright head roll test (UHRT) has been recently described in order to improve the diagnostic sensitivity of MSS (30). UHRT is performed in the upright position, and the patient' head is bent laterally toward one side, on the roll plane. This maneuver allows the gravity vector to move debris within LSC. Once horizontal nystagmus (either geotropic or apogeotropic) has been elicited, the head is slowly brought back to the center, and then the same procedure is repeated contralaterally (30).

UHRT has been conceived as a complementary test to HPT so that the affected side and LSC-BPPV variant could be determined from the upright position by matching nystagmus evoked by these two tests, sparing the patients troublesome symptoms.

**Figure 2:**
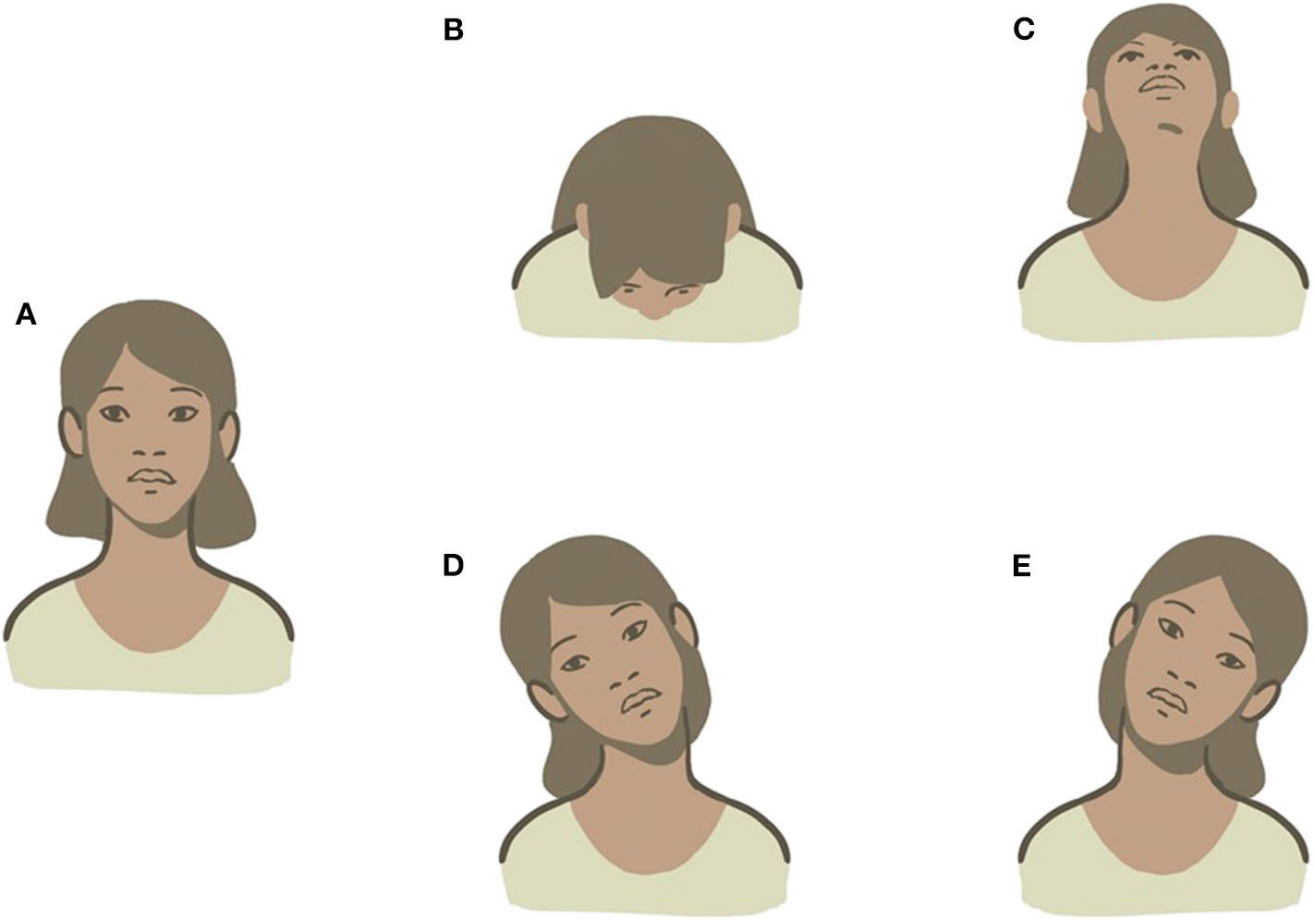
UBP for BPPV-LSC. **(A)** Detection of pseudo-spontaneous nystagmus (PSN). **(B)** Head pitch test (HPT) with forward head bending. **(C)** HPT with backward head bending. **(D)** Upright head roll test (UHRT) with rightward head tilting. **(E)** UHRT with leftward head tilting.

We combined the tests performed in the upright position (PSN, HPT, and UHRT) into a new diagnostic protocol named “upright BPPV protocol” (UBP), aiming to diagnose LSC-BPPV in the sitting position, causing the least possible discomfort to patients, like MSS. UBP is described in Figure 2.

The aim of this study is to determine the feasibility of UBP in the diagnosis of LSC-BPPV, comparing its outcomes with those obtained using the complete diagnostic protocol (CDP) including both upright and supine tests (PSN + HPT + UHRT + SSPT + SHYT). We also compared UBP results with those achieved with the supine diagnostic protocol (SDP, consisting in SSPT + SHYT) and the sole SHYT. Moreover, we aimed to check whether the concordance of correct diagnoses provided with UBP and CDP remains high over time, when diagnostic tests may likely produce partially incomplete or unclear results, compared to diagnoses provided with either SDP or with SHYT.

## Materials and Methods

The study was conducted in eight centers from June 2019 to February 2020 and was approved by the local ethics committees (approval number for the promoter institution: 237/2020/OSS/AUSLRE). All experimental procedures were performed in accordance with the Helsinki declaration and its amendments for human experimentation. Whereas, only one otoneurologist in each center was involved in patients' assessment and data collection, overall data were then analyzed by specialists of all 20 different institutions involved.

### Study Design

Chart review of adult patients diagnosed with LSC-BPPV was carried out. All patients enrolled in the study were evaluated with monocular or binocular video Frenzel goggles with a three-step diagnostic test battery according to the following order:

1. UBP: PSN, HPT, and UHRT
2. SSPT
3. SHYT

Collected clinical data included patient's personal information, time elapsed from symptom onset, direction of nystagmus observed during each maneuver, final diagnosis, and treatment performed with corresponding outcome.

Patients presenting with multiple semicircular canal involvement, with atypical forms of LSC-BPPV (i.e., canalith jam) or with coexistent vestibular disorders other than BPPV were excluded, as well as patients whose clinical chart was incomplete.

One hundred and thirty-four consecutive patients diagnosed with LSC-BPPV at different onset times were finally included in the study. Demographic data are summarized in Table 1. All data were systematically entered into a Microsoft Excel sheet (Microsoft Corporation, Redmond, Washington) shared by all centers involved in the study. Data were then collected and processed for statistical analysis.

**Table 1:** Demographic data.

	**Age**	**Onset time**		**Side**		**Geotropic forms**	**Apogeotropic forms**
62 Males (46.26%)	56.82 ± 14.46 (range: 25–89)	<48 h	21 (15.67%)	Right	33 (26.62%)	21 (15.67%)	12 (8.95%)
		2–7 days	23 (17.16%)				
		>7 days	18 (13.43%)	Left	29 (21.64%)	20 (14.92%)	9 (6.71%)
72 Females (53.73%)	57.04 ± 15.58 (range: 22–85)	<48 h	20 (14.92%)	Right	39 (29.10%)	22 (16.41%)	17 (12.68%)
		2–7 days	34 (25.37%)				
		>7 days	18 (13.43%)	Left	33 (26.64%)	17 (12.68%)	16 (11.94%)
	56.94 ± 15.01 (range: 22–89)				134 (100%)	80 (59.70%)	54 (40.29%)

### Upright BPPV Protocol

The first step in UBP was the detection of PSN, defined as a horizontal long-lasting, non-paroxysmal nystagmus observed with the patient in the sitting position, with his head in axis with his body, so that LSC is 30° inclined with respect to the horizontal plane (upright position). If detectable, PSN direction was reported.

The second step consisted of observing nystagmus patterns during HPT. This maneuver was performed by slowly bending the patient's head 60° forward and then 30° backward with respect to the horizontal plane. The head was held still up to 30 s in both positions until nystagmus appears. In LSC-BPPV, the nystagmus elicited by HPT is purely horizontal and changes direction according to head positions. The direction of the nystagmus was recorded in both positions. HPT was classified as negative (if no nystagmus was detected), positive incomplete (if nystagmus was observed in only one position), and positive complete (if nystagmus was evoked in both positions).

UHRT represented the third step in UBP. First, the head of the patient was slowly bent about 30° laterally toward one side, in the roll plane, bringing the patient's ear closer to the shoulder on the same side. The head was held still up to 30 s in this position until nystagmus appears. Then, the head was slowly brought back to the center and held upright for an additional 30 s, to allow the resulting endolymphatic flows to restore. Thereafter, the same maneuver was performed toward the contralateral side. The direction of the nystagmus was recorded in both positions. UHRT was classified as negative (if no nystagmus was detected), positive incomplete (in case it was recorded in only one side), and positive complete (if it was detected in both sides). UBP is described in Figure 2.

### SSPT

SSPT was performed quickly by bringing the patient from the sitting to the supine position and observing the resulting nystagmus. If nystagmus could be detected, its direction was recorded, indicating whether it was in accordance with the nystagmus evoked by SHYT.

### SHYT

SHYT was performed by turning the patient's head 180° to either side while supine. The direction of the nystagmus, either geotropic or apogeotropic, was recorded, specifying on which side the nystagmus was greater if asymmetry between positionings could be detected.

### LSC-BPPV Treatment and Outcome

Several therapeutic strategies were adopted for LSC-BPPV with significant differences among different institutions. All therapeutic techniques performed in each patient were recorded and sorted according to the order of execution.

Therapeutic outcome was assessed by SHYT in the same session, 10–30 min after physical therapy or during a follow-up examination after 24–72 h, depending on protocols in use in the different centers and on patients' compliance. Outcomes were classified into resolution, resolution following conversion, failure, and unknown.

LSC-BPPV was considered as resolved if no nystagmus was evoked at the last follow-up SHYT, specifying whether a conversion in another form of BPPV occurred before resolution (i.e., conversion from apogeotropic to geotropic forms of LSC-BPPV or from LSC-BPPV to posterior semicircular canalolithiasis). Persistence of positional nystagmus at the second follow-up evaluation was classified as treatment failure. Outcome was classified as unknown if the patient did not attend the follow-up examination.

### Statistical Analysis

Continuously distributed variables were described by median, mean, and SD; categorical variables were described by frequencies and percentages.

A chi-square test and Cohen's kappa statistics were performed to compare concordance between different protocols results. Thresholds of significance level were set to 0.05. Analyses have been processed using Sklearn, SciPy, and Pandas libraries in Python code (Python Software Foundation).

## Results

Clinical charts from 134 patients (62 males, 72 females, age: 22–89 years, mean 56.94 ± 15.01 years) with LSC-BPPV were considered for this study.

The time between symptom onset and diagnosis was <48 h in 41 patients (30.59%), ranged from 2 to 7 days in 57 cases (42.53%), and exceeded 7 days in 36 cases (28.86%), without significant differences according to age, involved ear, and LSC-BPPV form.

CDP was considered as reference for diagnosis (gold standard). The right side was involved in 72 cases (53.73%); canaliths were located in the non-ampullary arm of LSC in 43 cases (59.72%), whereas otoconial debris were in the ampullary arm in the remainder (40.27%). The left ear was affected in 62 patients (46.26%); geotropic forms were diagnosed in 37 cases (59.67%) and apogeotropic variants in 25 cases (40.32%). No differences were observed according to age, gender, and onset time.

PSN could not be detected in 63 cases (47.01%), whereas it always matched with the nystagmus detected in HPT with backward head bending in the remaining cases. HPT was classified as positive complete in 93 cases and positive incomplete in 35 (69.4 and 26.11%, respectively), whereas no nystagmus was detected in six subjects (4.47%). UHRT was positive in 133 (99.25%) cases (27 positive incomplete, 20.14%). Either UHRT or HPT was positive but incomplete in 50 cases (37.31%), while in 12 cases (8.85%), both tests were classified as positive incomplete, without statistical differences for age, gender, and affected side.

Associations between onset time and presence/absence of PSN and positivity for both HPT and UHRT tests were evaluated, but no statistically significant results were achieved. Only detectable PSN and onset time analysis achieved a significant *p*-value (0.014), but the strength of association was extremely weak (Cramer's *V* = 0.0637).

Nystagmus was observed with SSPT in 109 patients (81.34%), whereas this test was negative in 25 (18.65%) subjects, mostly presenting with apogeotropic forms (76%, *p* < 0.001). Furthermore, in five patients (4.48%), the direction of nystagmus did not comply with the nystagmus evoked by SHYT.

SHYT allowed diagnosis in 116 patients (86.56%), whereas in 18 (13.43%), it only allowed identification of the LSC-BPPV form despite failing to detect the affected side. Diagnosis was mainly missing in cases with apogeotropic variants (16 cases, 88.88%, *p* < 0.001). There were no statistically significant differences for age, gender, and involved side.

UBP protocols led to the same diagnosis obtained using CDP in 128 patients (95.5%). Statistical concordance between these protocols was significant (*p* < 0.000), as shown by high values of chi-square (376.4) and Cohen's kappa (0.94). CDP and UBP continued to show statistically significant concordant diagnosis even when analyzing cases in which HPT and UHRT provided positive incomplete outcomes, as shown in Table 2.

**Table 2:** Concordance between complete diagnostic protocol (CDP) and upright BPPV protocol (UBP) with positive incomplete outcomes of head pitch test (HPT) and upright head roll test (UHRT).

**Cases**	**No. of cases**	**% concordance**	**Chi-square test**	***p*-value**	**Cohen's kappa**
Incomplete HPT	35	100.0%	105.0	<0.000	1.000
Incomplete UHRT	27	96.3%	81.0	<0.000	0.947
HPT and UHRT both incomplete	12	100.0%	36.0	<0.000	1.000

Similar outcomes could be found when comparing CDP and SDP outcomes, despite a weaker strength of relationship. CDP and SDP protocols provided identical diagnosis in 85.1% of cases (114 on 134), with statistically significant concordance (chi-square = 339.0, Cohen's kappa = 0.84, *p* < 0.000).

SHYT provided statistically significant concordant results (*p* < 0.000) with CDP (85.1% of cases), not lower than the results obtained using SDP.

In our series, 119 patients (88.80%) were successfully treated within two sessions with physical therapy. In 26 of them (21.84%), LSC-BPPV was converted into another BPPV form before resolution, whereas two patients (1.49%) did not attend the scheduled follow-up evaluation and therapeutic outcome was not assessed. In 13 subjects (9.70%) LSC-BPPV was not resolved within two sessions, and these cases were classified as “treatment failure.” Among them, the apogeotropic form was diagnosed in 12 cases (92.03%).

The canalith repositioning procedure according to Gufoni et al. (31) was the most frequently used technique as first-line therapy, being applied in 65 patients presenting with geotropic variants (81.25%) and in 32 with apogeotropic variants (59.25%).

## Discussion

All tests proposed for the diagnosis of BPPV, regardless of the involved canal, are based on head movements on different planes of the space. With head movement, otoconial debris can gravitate within the semicircular canals, eliciting ocular movements or modifying underlying ongoing nystagmus. In light of the above, diagnostic tests can be properly described according to the axis around which the head moves and to patients' position (upright or supine).

**Figure 3:**
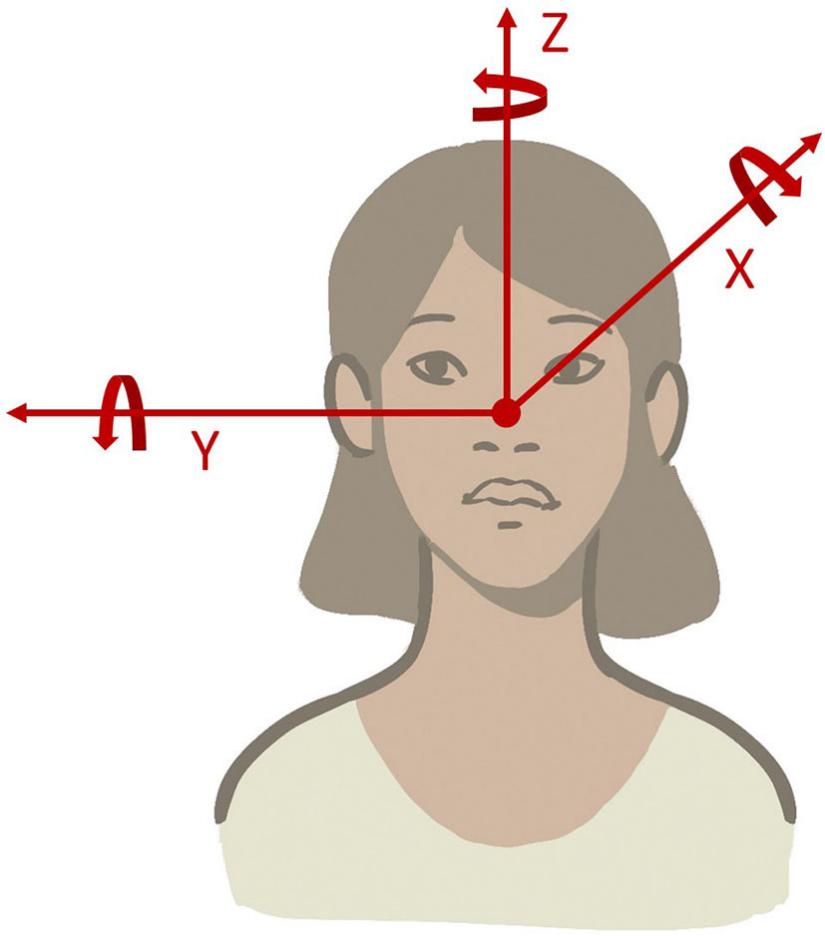
Schematic overview of head rotations along three axes (X, Y, and Z). Axes are defined relative to the person, not to gravity.

The head can rotate around the X (roll), Y (pitch), and Z (yaw) axes originating at the intersection of the midsagittal plane with the interocular axis (the nasion) (Figure 3). Consequently, the following head movements were found:

- Head movements in the yaw plane (i.e., around the rostral-caudal, yaw, or z-axis) are horizontal.
- Head movements in the pitch plane (i.e., around the inter-aural, pitch, or y-axis) are vertical.
- Head movements in the roll plane (i.e., around the naso-occipital, roll, or x-axis) are torsional.

Rotations around these axes can be performed slowly or rapidly. In the first case, debris are moved only by gravity, whereas inertia of otoconial fragments adds up to the gravitational vector if movements are brisk.

Diagnosis of LSC-BPPV is traditionally based on the features of nystagmus elicited by SHYT, which is performed in the supine position and evokes a direction-changing horizontal nystagmus according to head rotations around the z-axis. The nystagmus herein elicited may be paroxysmal or persistent, beating toward the ground (geotropic) or in the opposite direction (apogeotropic). The nystagmus direction suggests the position of debris that may settle in the short arm, close to the cupula, or freely float in the non-ampullary arm. Diagnosis of the involved side (lateralization) can be achieved by comparing the intensity of the nystagmus evoked on each side. Nevertheless, determining the affected ear based on Ewald's second law and related asymmetrical outputs may be challenging in clinical practice, mostly because the intensity and amplitude of eye movements may be symmetrical. Our data showed that SHYT is unable to determine the involved side in 13.43% of patients, consistent with other studies aiming to define accuracy of lateralization in LSC-BPPV (14, 32).

In our study, most patients with non-diagnostic SHYT were affected by apogeotropic forms, exhibiting small-amplitude direction-changing nystagmus leading to hardly comparable responses on either side. This finding matched with the mathematical model proposed by Squires et al. suggesting that canalithiasis represents a mechanism likely stronger than cupulolithiasis in deflecting ampullary cupula. Therefore, a bigger amount of debris or larger otoconia is necessary to produce the same nystagmus intensity in cupulolithiasis as that in canalithiasis (33, 34).

Although sensitivity of SHYT may be improved using a video-oculography system, this technology is not always available in clinical practice. On the other hand, repeating the test to confirm the diagnosis may result in impaired paroxysmal nystagmus due to fatigue response and significant discomfort for patients with acute vertigo and intense autonomic symptoms (14, 29).

However, other findings may provide clues to determine the affected ear in LSC-BPPV without comparing intensities of direction-changing positional nystagmus with SHYT. PSN represents the easiest finding among secondary signs of lateralization as it can be observed directly in the neutral sitting position (14–19, 29). In fact, LSC acts as an inclined surface drawing a 30° front-open-angle with the horizontal plane, allowing otoconial debris to move along the canal (in canalolithiasis) or resulting in a persistent cupular displacement (in cupulolithiasis). Therefore, PSN, when detectable, is directed to the healthy side in geotropic forms and to the affected side in apogeotropic variants (13, 15–18). Although its pathophysiology is not yet fully understood, a long-lasting course of PSN elicited in both cases has been supposed to result from the action of different forces exhibiting similar amplitudes though acting in opposite directions on the otolith mass: gravity, which moves otoliths along the LSC, balanced by fluid viscosity and endolymphatic friction (15).

In our series, PSN was detectable in 53.09% of cases, mostly among patients with recent onset of symptoms. This finding suggested that disaggregation of the original, heavy otoconial cluster represents a time-dependent phenomenon occurring spontaneously and resulting in dispersion of several fragments. Therefore, otoconial debris dispersed along the canal and, adherent to walls of membranous canals, could become “silent” over time, namely, unable to induce cupular deflection in the absence of head movements (34).

Changes in PSN direction occur when performing HPT, consisting of neck flexion and extension. PSN usually disappears by bending the head 30° forward as LSC reaches a neutral position, almost parallel to the ground. Bending the head further 30° forward results in an ampullopetal endolymphatic flow in geotropic forms, accounting for nystagmus toward the affected side while accounting for an ampullofugal flow in apogeotropic BPPV, resulting in nystagmus toward the healthy side. An opposite endolymphatic flow could be obtained by bending the head 60° backward, resulting in a horizontal nystagmus toward the healthy side in geotropic forms and toward the affected side in apogeotropic variants (14, 15, 20–22, 29).

Our data show that the nystagmus evoked by HPT represents an almost constant finding, providing highly reliable information for lateralization. However, HPT alone does not allow clinicians to identify which LSC and canal arm are involved. Nevertheless, it allows us to restrict the diagnostic hypotheses to only two options: the geotropic variant of one side or apogeotropic variant involving the opposite LSC (30).

Considering a hypothetical case with LSC-BPPV as a practical example, evaluation of a patient in the upright position presenting with left-beating horizontal PSN can be assumed. When the patient's head is bent forward, the nystagmus first disappears and then reverses, becoming right beating. Moreover, when the head is bent backward, the nystagmus changes its direction returning to being left beating. In this case, only two options are possible: right geotropic LSC-BPPV or left apogeotropic LSC-BPPV.

Although it has been described how the evaluation of nystagmus intensity by moving the head on the pitch plane might distinguish geotropic from apogeotropic forms, identification of otolith location with only HPT is challenging in most cases (22).

Nystagmus evoked by UHRT appeared as a reliable lateralization sign in almost all cases, so that CDP and UBP protocols led to the same diagnosis in 95.5% of cases with extremely high levels of concordance, even higher than those achieved by SDP and SHYT alone. In UBP, indeed, UHRT is performed sequentially after HPT, aiming to complete the diagnostic workup in the sitting position, by simply tilting the patient's head sideways along the roll plane and observing the direction of nystagmus (geotropism) (30).

**Figure 4:**
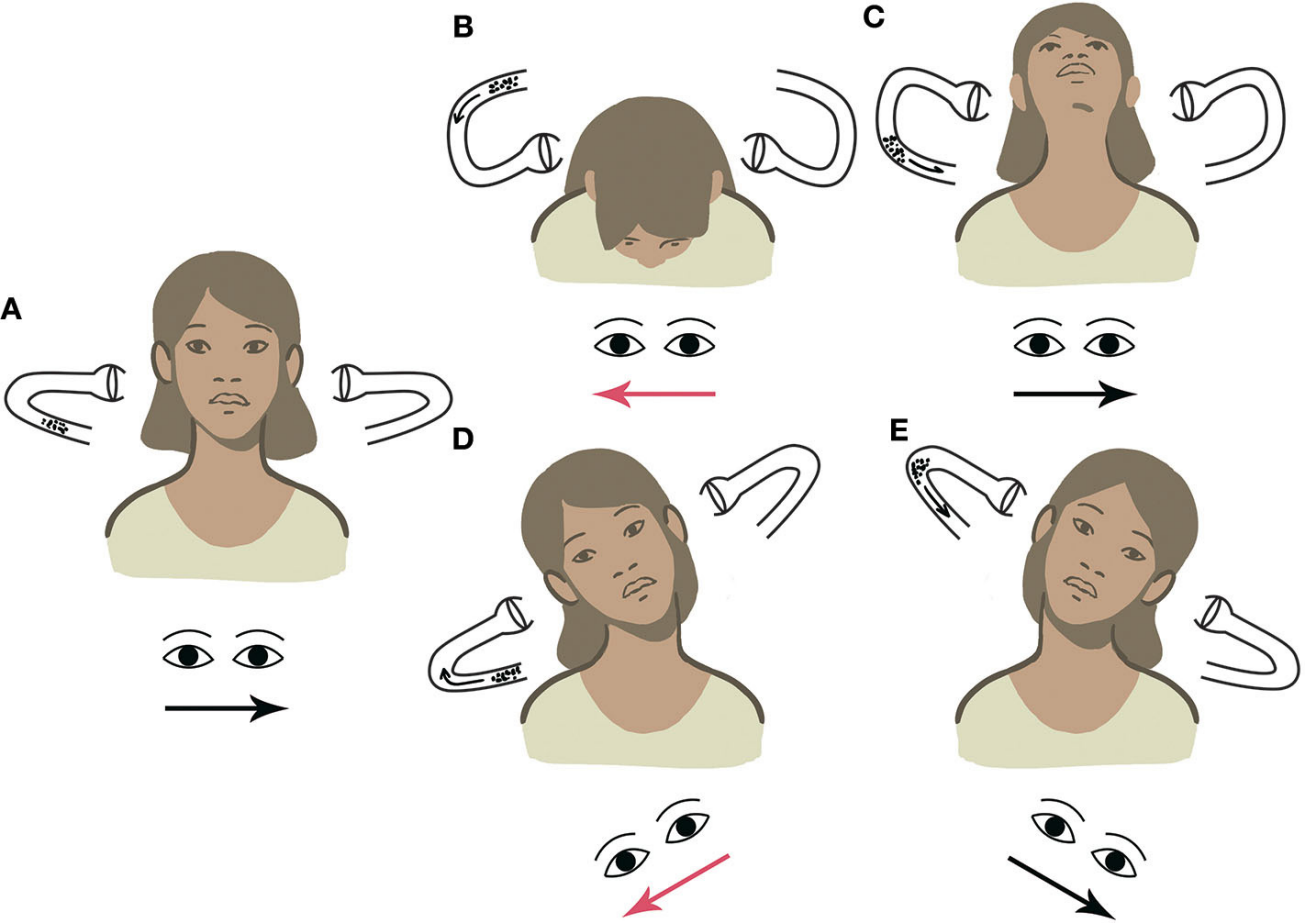
UBP for right geotropic LSC-BPPV. Arrows within the canal represent the direction of endolymphatic flows, whereas arrows beneath the eyes represent the direction of the fast phase of nystagmus. Right-beating nystagmus is represented in red. **(A)** PSN: left beating. **(B)** HPT with forward head bending: right-beating nystagmus. **(C)** HPT with backward head bending: left-beating nystagmus. **(D)** UHRT with rightward head tilt: right-beating geotropic nystagmus. **(E)** UHRT with leftward head tilt: left-beating geotropic nystagmus.

**Figure 5:**
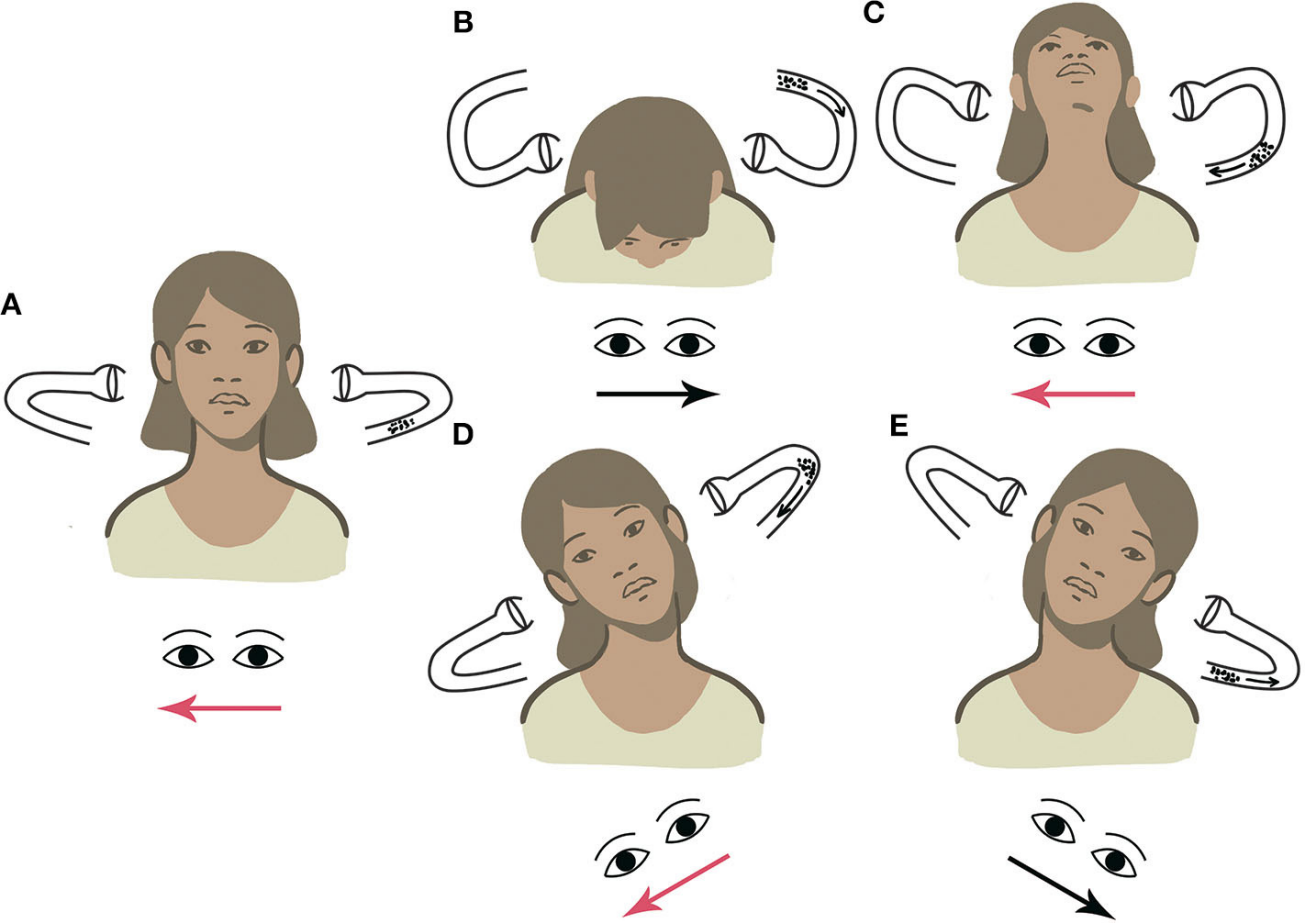
UBP for left geotropic LSC-BPPV. **(A)** PSN: right beating. **(B)** HPT with forward head bending: left-beating nystagmus. **(C)** HPT with backward head bending: right-beating nystagmus. **(D)** UHRT with rightward head tilt: right-beating geotropic nystagmus. **(E)** UHRT with leftward head tilt: left-beating geotropic nystagmus.

**Figure 6:**
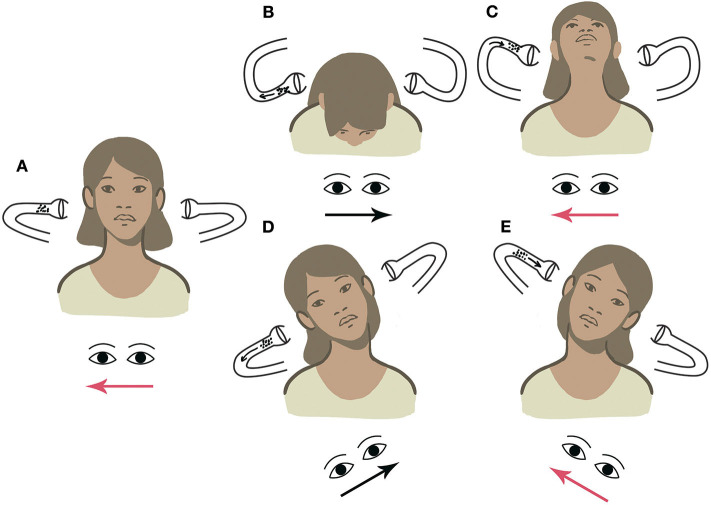
UBP for right apogeotropic LSC-BPPV. **(A)** PSN: right beating. **(B)** HPT with forward head bending: left-beating nystagmus. **(C)** HPT with backward head bending: right-beating nystagmus. **(D)** UHRT with rightward head tilt: left-beating apogeotropic nystagmus. **(E)** UHRT with leftward head tilt: right-beating apogeotropic nystagmus.

**Figure 7:**
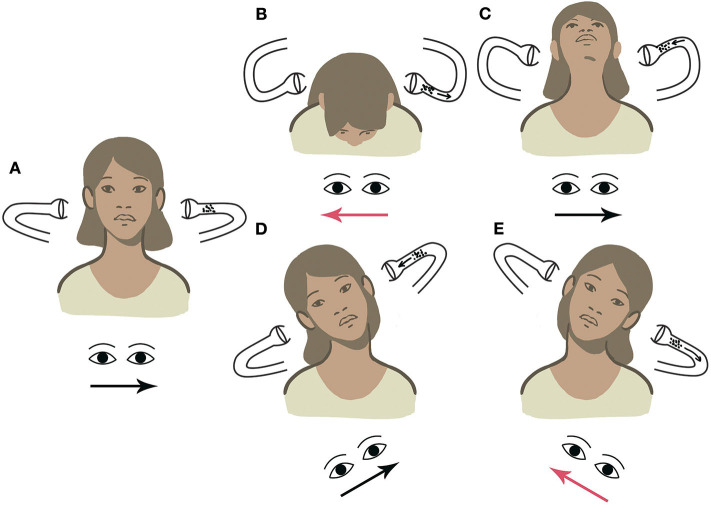
UBP for left apogeotropic LSC-BPPV. **(A)** PSN: left beating. **(B)** HPT with forward head bending: right-beating nystagmus. **(C)** HPT with backward head bending: left-beating nystagmus. **(D)** UHRT with rightward head tilt: left-beating apogeotropic nystagmus. **(E)** UHRT with leftward head tilt: right-beating apogeotropic nystagmus.

Considering the above-mentioned example, if left-beating nystagmus (apogeotropic) is elicited by tilting the head toward the right, debris can be easily localized within the ampullary arm of left LSC, consistent with left apogeotropic LSC-BPPV. When the patient's head is tilted toward the contralateral side, apogeotropic nystagmus (right beating) could be likely evoked again, thus confirming the diagnostic hypothesis (Supplementary Video 1). On the contrary, if geotropic nystagmus is elicited with UHRT on either side, otoliths should be considered as settling in the non-ampullary arm of the right LSC, thus allowing us to diagnose right geotropic LSC-BPPV (Supplementary Video 2). Other examples of LSC-BPPV diagnosed using UBP are reported in Supplementary Videos 3, 4. Figures 4–7 summarize how the nystagmus direction changes in relation to head positions with UBP in all possible four scenarios.

Although a first algorithm for nystagmus evaluation in the upright position was proposed by Frenzel (35), the use of tests in the sitting position for the diagnosis of BPPV is not widespread among clinicians. However, the opportunity to define the side and form of LSC-BPPV by only relying on maneuvers in the upright position may improve the tolerability of the diagnostic workup for patients, especially in the acute stage of the disease when they could be particularly susceptible to rotational movements and accelerations. Furthermore, UBP could also result in a less time-consuming management of LSC-BPPV as patients can directly receive appropriate treatment from the upright position, immediately after diagnosis. The repositioning procedure proposed by Gufoni, which was the treatment of choice in our series, starts indeed with the patient in the sitting position with his legs out of the examination couch (31).

Although simple and easy to perform, UBP may be challenging in patients with reduced cervical range of motion, similar to other maneuvers for BPPV (36). Nevertheless, should the patient exhibit difficulties in head extension/flexion or in lateral tilting due to neck stiffness, his whole trunk may be slightly bent about 30° along the pitch or the roll planes to attain the same head positions with respect to gravity, keeping the diagnostic value of UBP unchanged (30).

As often observed in our series, UBP may produce incomplete responses since several factors could determine the lack of nystagmus during upright tests. In physiological conditions, the most relevant factors affecting detection of nystagmus during diagnostic maneuvers for BPPV are the plane aligning with the movement performed, imprinted accelerations, otolith size, and location. In fact, whereas larger fragments should float more quickly within the endolymph, producing more intense nystagmus than small-sized debris, interactions between canaliths and canal walls could also likely account for the considerable variability in duration and latency of nystagmus, assuming that debris settling closer to canal walls should result in less intense endolymphatic flows and nystagmus (33, 34). Then, the same diagnostic test could result in different outcomes according to the features of otoconial cluster, thus evoking weaker and long-latency nystagmus if small particles are dispersed along the canal walls, while accounting for more intense and prompt nystagmus if a single clumped stone floats in the canal lumen.

Although both HPT and UHRT, unlike SHYT, have been conceived for exploiting gravity to move otoconial fragments along LSC and elicit nystagmus, the sole gravitational vector may not be effective enough to displace the cupula in each position during UBP. Conversely, angular accelerations used in SHYT may likely break canal wall interactions mobilizing canaliths, thus explaining the reason that this test results in detectable nystagmus in most LSC-BPPV (33, 34).

Should nystagmus be missing at the upright tests, clinicians may increase UBP sensitivity by imparting slight accelerations to the patient's head by moving it quickly from a position to the other along the roll or the pitch plane. This way, inertial forces will likely help the gravity vector to generate endolymphatic flows, resulting in detectable nystagmus (30).

Nevertheless, according to our findings, proper diagnosis of LSC-BPPV could be achieved even if UBP test battery gives incomplete results, as shown in Supplementary Video 4. Theoretically, even if PSN was not detected, the side and form of LSC-BPPV could still be properly identified with at least only one nystagmus evoked each by HPT and UHRT.

Since nystagmus resulting from HPT and UHRT are often weak, the major limitation of UBP is differentiating BPPV subtypes with paroxysmal nystagmus (canalolithiasis) from variants with persistent nystagmus (cupulolithiasis). This aspect may be relevant in cases with direction-changing positional nystagmus due to central disorders mimicking BPPV, such as vestibular migraine (37, 38). Therefore, if clear nystagmus is not observed at least in one position for HPT and UHRT or in cases with atypical clinical history, diagnostic tests in the supine position are strongly recommended.

In addition to SHYT, SSPT was described to contribute to lateralization according to LSC geometry, as the canal plane changes alignment from about horizontal to vertical when the head moves from the sitting to supine positions. In apogeotropic variants, debris in the short arm of LSC cause ampullopetal deflection resulting in horizontal nystagmus toward the affected side. Conversely, in geotropic forms, canaliths within the non-ampullary arm move away from the cupula, eliciting an ampullofugal deflection and nystagmus toward the healthy side (26, 27).

Although SSPT may help clinicians to discriminate the involved ear, its sensitivity appears controversial (26, 39). Accordingly, in our series, this test did not lead to nystagmus in 18.65% of patients, and its lateralization rates were significantly lower in apogeotropic cases compared to geotropic forms. As discussed previously, these findings may be explained by the lower responsivity of LSC when otoconial matter is located in the short arm. Nevertheless, in our series, upright tests were scheduled as first tests according to the protocol (hence performed prior to supine maneuvers) and might have impaired SSPT sensitivity by dispersing canaliths along canals and reducing their “piston action” on the ampullary cupula (40). Finally, in our study, SDP and SHYT alone provided the same diagnostic concordance with CDP, indicating the limited role of SSPT in defining the diagnosis for LSC-BPPV.

Being a retrospective multicenter study, conclusions of our analysis present important limitations. They mainly include that it is not possible to ensure that observation of nystagmus has always been performed under the same conditions across all involved institutions, although only one otoneurologist in each center was involved in patients' assessment and data collection. Therefore, our data need to be confirmed by further studies with a prospective design and a common protocol shared by a larger amount of centers including larger cohorts.

## Conclusions

According to our results, LSC-BPPV diagnosis can be obtained in the sitting position with upright diagnostic tests. Furthermore, UBP is a reliable algorithm to diagnose LSC-BPPV, and our detailed explanation of maneuvers proves that this study can be reproduced without difficulty. Then, in line with MMS principles, UBP can likely spare patients unpleasant maneuvers, allowing clinicians to proceed immediately with proper physical treatment. Nevertheless, SHYT is still required if oculomotor findings in the upright position are lacking or unclearly detectable or in cases where other vestibular disorders may mimic LSC-BPPV presenting with direction-changing positional nystagmus.

Further investigations following a prospective study, involving more centers, and including lager cohorts will be needed to determine the sensitivity of UBP in detecting otolith location in LSC-BPPV.

## Data Availability Statement

The original contributions presented in the study are included in the article/Supplementary Materials, further inquiries can be directed to the corresponding author/s.

## Ethics Statement

The studies involving human participants were reviewed and approved by Area Vasta Nord Emilia Romagna Institutional Review Committee (approval number: 237/2020/OSS/AUSLRE). The patients/participants provided their written informed consent to participate in this study. Written informed consent was obtained from the individual(s) for the publication of any potentially identifiable images or data included in this article.

## Author Contributions

SM, PM, and AC led the conception of the study, conducted most data acquisition and interpretation, and made significant contributions to the writing and editing of the manuscript, they are designated co-first authors for this study. RP, VM, ASc, GA, and GAL equally contributed in data acquisition. SM conducted data analysis and creation of figures. BG, MR, VM, and GAL were involved in manuscript editing and review. All authors involved equally contributed to data interpretation and manuscript review, and approved the final version of the manuscript.

## Conflict of Interest

The authors declare that the research was conducted in the absence of any commercial or financial relationships that could be construed as a potential conflict of interest.

## Supplementary Material

Supplementary Video 1 UBP for left apogeotropic LSC-BPPV.

Supplementary Video 2 UBP for right geotropic LSC-BPPV.

Supplementary Video 3 UBP for right apogeotropic LSC-BPPV.

Supplementary Video 4 UBP for left geotropic LSC-BPPV.

## Abbreviations

BPPV: benign paroxysmal positional vertigo
CDP: complete diagnostic protocol (PSN + HPT + UHRT + SSPT + SHYT)
HPT: head pitch test
LSC: lateral semicircular canal
MSS: minimum stimulus strategy
PSN: pseudo-spontaneous nystagmus
SDP: supine diagnostic protocol (SSPT + SHYT)
SHYT: supine head yaw test
SSPT: seated-supine positioning test
UBP: upright BPPV protocol (PSN + HPT + UHRT)
UHRT: upright head roll test.
